# Is it acceptable to approach colorectal cancer patients at diagnosis to discuss genetic testing? A pilot study

**DOI:** 10.1038/sj.bjc.6601332

**Published:** 2003-10-14

**Authors:** M Porteous, M Dunckley, S Appleton, S Catt, M Dunlop, H Campbell, A Cull

**Affiliations:** 1 Department of Clinical Genetics, Molecular Medicine Centre, Western General Hospital, Crewe Road South, Edinburgh EH4 2XU, UK; 2 Cancer Research UK, Edinburgh Oncology Unit, Western General Hospital, Crewe Road South, Edinburgh EH4 2XR, UK; 3 Colon Cancer Genetics Group and Academic Coloproctology, MRC Human Genetics Unit, Western General Hospital, Crewe Road South, Edinburgh EH4 2XU, UK; 4 Department of Public Health Sciences, University of Edinburgh Medical School, Teviot Place, Edinburgh EH8 9AG, UK

**Keywords:** colorectal cancer patients, genetic testing, hereditary nonpolyposis colorectal cancer, acceptability

## Abstract

In this pilot study, the acceptability of approaching 111 newly diagnosed colorectal cancer patients with the offer of genetic testing for hereditary nonpolyposis colorectal cancer (HNPCC) was assessed. A total of 78% of participants found it highly acceptable to have the information about HNPCC brought to their attention at that time.

Although most cases of colorectal cancer occur sporadically, a small proportion are due to known hereditary syndromes where the onset of disease is at an earlier age. The commonest of these is hereditary nonpolyposis colorectal cancer (HNPCC), an autosomal dominant syndrome resulting from mutations in mismatch repair (MMR) genes, which accounts for less than 1% of all colorectal cancers (Evans *et al*, 1997). It has been estimated that male carriers of an MMR mutation have a 74% lifetime risk of developing colorectal cancer compared to 30% in females, who also have a 42% risk of endometrial cancer (Dunlop *et al*, 1997).

The Colorectal Cancer Genetic Susceptibility (COGS) study aims to evaluate a new Scottish-wide research strategy for identifying individuals at high risk of developing HNPCC. Newly diagnosed colorectal cancer patients under the age of 55 years are offered genetic testing for three mutations in MMR genes (i.e. MLH1, MSH2, MSH6). This allows the identification of HNPCC families and enables appropriate screening to be offered to asymptomatic relatives at increased risk of the disease and to index cases postsurgery.

Although a number of studies have investigated the psychosocial aspects of genetic testing for HNPCC in colorectal cancer patients (Vernon *et al*, 1997; Gritz *et al*, 1999; Kinney *et al*, 2000; Esplen *et al*, 2001; Keller *et al*, 2002; Hadley *et al*, 2003), little is known about the acceptability of approaching newly diagnosed colorectal cancer patients with the offer of genetic testing.

This paper reports descriptive results of the pilot phase of a patient feedback study. This study was an integral part of the COGS study aiming to determine whether it was acceptable to approach recently diagnosed colorectal cancer patients about genetic testing at a time when they were already adjusting to a new diagnosis.

## MATERIALS AND METHODS

### Participants

Ethical approval for the COGS study was obtained from the multicentre research ethics committee. Eligible patients in Lothian and Greater Glasgow who were approached for the COGS study (with the permission of their health-care professional), were invited to participate in the patient feedback study regardless of whether they decided to participate in the COGS study but provided that they:

1. had received a home visit from their local study recruitment nurse.
2. were willing to be contacted to discuss the reasons for their decision to/not to participate in the COGS study.

### Procedure

Patients were invited to participate in the COGS study (pre- or postoperatively) by their specialist nurse or by post. All patients expressing interest were visited in their own home by the local study recruitment nurse to: discuss the COGS study; provide genetic counselling by constructing a detailed pedigree and producing an estimated risk of HNPCC for their relatives (low/moderate/high) based on Scottish guidelines (Haites *et al*, 2000); gain written consent; take a blood sample for genetic testing. At the end of the visit, all patients meeting the patient feedback study criteria were given an information sheet about the study and a feedback questionnaire, which they were asked to return in the freepost envelope provided.

### Measures

Participants' age, gender and the risk of HNPCC to their relatives were derived from clinical records.

The brief feedback questionnaire consisted of several study-specific items (with multiple choice or Likert scale responses) to assess:

1. Sociodemographic characteristics (i.e. marital status, education, number of children).
2. Prior awareness of familial cancer risk.
3. Acceptability of approach with genetic information.
4. Decision-making factors concerning the genetic test.
5. Subjective understanding of issues discussed in genetic counselling.
6. Expectation of the genetic test result.
7. Cancer and genetics worry.

## RESULTS

A total of 160 patients, who were approached to participate in the COGS study between February 1999 and July 2000, were eligible to participate in the patient feedback study. Of the 160 questionnaires given to patients, 111 (69%) were returned completed. Only one of the 111 patients who completed the questionnaire had declined to participate in the COGS study.

Of the 111 participants, 61 (55%) were male and 50 (45%) were female ranging in age from 31 to 55 years (mean=48.6, s.d.=5.5). The risk of HNPCC for their relatives was low for 60% of participants (*n*=65), moderate for 39% (*n*=42) and high for 2% (*n*=2). The majority were married or living with a partner (*n*=90, 83%), and had been educated up to age 16 years only (*n*=56, 60%). In all, 81% (*n*=89) had at least one child.

Most participants (*n*=61, 58%) had no prior awareness that colorectal cancer could run in families. A total of 36% (*n*=37) had been concerned that colorectal cancer might run in their family. Of the 31% (*n*=33) who had any experience of colorectal cancer in a relative, 50% (*n*=15) had been aware that colorectal cancer could run in families and 69% (*n*=20) had been concerned about this.

In all, 78% (*n*=87) thought it was definitely acceptable to have this genetic information brought to their attention at that time and the remainder (*n*=24, 22%) did not mind being approached with the information.

After receiving the information sheet about the COGS study, but prior to discussing participation with a member of staff, 91% (*n*=101) reported that they had decided to take the genetic test, one individual (1%) had decided not to take the test and nine (8%) were undecided.

Table 1

**Table 1:** Importance of several factors in participants' decision-making about genetic testing

	**Frequency of ratings on level of importance**		
**Decision-making factor**	**Very**	**Somewhat**	**Not**
*Potential advantages*			
To learn about children's risk^b^	87 (97%)	2 (2%)	1 (1%)
To help research	99 (89%)	10 (9%)	2 (2%)
Access to screening for family	94 (85%)	14 (13%)	3 (3%)
To inform follow-up care	90 (82%)	15 (14%)	5 (5%)
To learn more about own cancer	80 (73%)	23 (21%)	7 (6%)
To be reassured	74 (67%)	30 (27%)	7 (6%)
To plan for the future	70 (63%)	31 (28%)	10 (9%)
			
*Potential disadvantages*			
Concern about effect on family	95 (86%)	14 (13%)	2 (2%)
Concern that result not accurate	37 (34%)	36 (33%)	37 (34%)
Concern about causing problems, for example, insurance	31 (29%)	42 (39%)	36 (33%)
Concern with getting upset	29 (26%)	35 (32%)	46 (42%)

a Sample size varies due to missing data.

b Not applicable for the 21 participants who did not have children.

shows how participants rated the importance of several factors in their decision whether to undergo genetic testing. For all participants (except those without children), the decision-making factor most frequently rated as very important was ‘to learn about their children's risk of developing colorectal cancer'.

Participants' subjective understanding of issues discussed in genetic counselling is presented in Table 2

**Table 2:** Subjective understanding of issues discussed in genetic counselling

	**Frequency of ratings on understanding**	
**Issue discussed in genetic counselling**	**Understand**	**Not sure**
Why you have been offered the blood test?	111 (100%)	0
What the blood test is for?	109 (100%)	0
What a positive result would mean for you?	98 (92%)	9 (8%)
What a negative result would mean for you?	102 (95%)	5 (5%)
What a positive result would mean for your family?	100 (93%)	8 (7%)
What a negative result would mean for your family?	98 (93%)	8 (8%)

a Sample size varies due to missing data.

. The vast majority of participants felt that they had understood these issues.

A minority of participants (*n*=8, 7%) expected that they would receive a positive test result (i.e. they would carry the genetic mutation), 21% (*n*=23) expected to test negative and 72% (*n*=79) were unsure.

Most participants (*n*=97, 87%) reported some degree of difficulty (a little, quite, very) coming to terms with the diagnosis of cancer, with 15% (*n*=17) overall reporting that it had been very difficult. Similarly, the majority of participants (*n*=89, 80%) indicated that it had been difficult to cope physically with their illness and treatment, with eight participants (7%) overall reporting that it had been very difficult. A total of 45% of participants (*n*=48) rated their current worry about their cancer and its treatment at or above the midpoint of 4 on a 1 (not at all) to 7 (all the time) scale.

In total, 19% of participants (*n*=21) rated their current level of worry caused by the genetics information at or above the midpoint of 4 on a 1 (not at all) to 7 (all the time) scale. Of these 21 participants, 80% (*n*=16) had rated their current worry about their cancer and its treatment at or above the midpoint, 57% (*n*=12) and 43% (*n*=9) had been informed that their relatives were at low or moderate risk of HNPCC, respectively.

## DISCUSSION

The majority of participants found it highly acceptable to have information about HNPCC brought to their attention at a time when they were coping with a new diagnosis of colorectal cancer, despite a lack of prior awareness that the disease could run in families. The vast majority had decided to accept the offer of a genetic test and reported high levels of subjective understanding concerning genetic testing. Similar findings have been reported in American colorectal cancer patients in terms of positive attitudes to undergoing HNPCC genetic testing (Gritz *et al*, 1999).

The results suggest that although receiving information about HNPCC did not cause most participants undue worry, a minority of participants rated this worry at the top end of the scale. This was despite the fact that most had been informed that their relatives were at low risk of HNPCC. Likewise, these participants tended to have rated worry about their cancer and its treatment at the top end of the scale. Further research is needed using standardised measures of psychological distress to assess the clinical significance of this finding among patients who may already be experiencing high levels of distress as a consequence of their recent diagnosis of cancer. This would enable appropriate psychological treatment to be offered to clinically distressed patients and interventions to be targeted at those patients who are likely to become distressed as a result of receiving information about HNPCC. The results of earlier studies of colorectal cancer patients undergoing genetic testing suggested that 17% (Gritz *et al*, 1999) to 24% (Vernon *et al*, 1997) were suffering from clinically significant levels of depression.

Further research is warranted to: investigate the reasons for nonparticipation in a study offering colorectal cancer patients genetic testing for HNPCC; identify characteristics that may help to explain why some patients are less likely to participate in this type of study; establish whether subjectively reported understanding of genetic testing can be objectively verified; assess the longer-term psychological consequences of this novel screening strategy.

Results from this pilot study will direct the assessment of a larger cohort of colorectal cancer patients invited to participate in the COGS study. The results of this subsequent research could help to determine whether this novel screening strategy has the potential to be incorporated into future NHS services for colorectal cancer patients.
